# Editorial: Epigenetics of B Cells and Antibody Responses

**DOI:** 10.3389/fimmu.2015.00656

**Published:** 2016-01-11

**Authors:** Hong Zan, Paolo Casali

**Affiliations:** ^1^Department of Microbiology and Immunology, University of Texas School of Medicine, UT Health Science Center, San Antonio, TX, USA

**Keywords:** AID, B cell, class switch DNA recombination, epigenetics, immunoglobulin, memory B cell, plasma cell, somatic hypermutation, V(D)J recombination

**The Editorial on the Research Topic**

**Editorial: Epigenetics of B Cells and Antibody Responses**

Epigenetics is the study of changes in gene activity that are heritable but not caused by changes in the DNA sequence. By modulating gene activities, epigenetic changes regulate cell functions. They include DNA methylation, histone post-translational modifications, and gene silencing by the action of non-coding RNAs, particularly microRNAs. It is now clear that epigenetic changes regulate B cell development. By acting in concert with networks of transcription factors, they modulate the activation of B cell lineage-specific gene programs and repress inappropriate gene transcription in particular B cell differentiation states (1).

The hallmark of B cell development in the bone marrow is the assembly of the B cell receptor (BCR) for antigen through rearrangement of immunoglobulin heavy (IgH) and light (IgL) chain V(D)J genes, as mediated by RAG1/RAG2 recombinases. Ig V(D)J rearrangement critically times the progression from pro-B cell to pre-B cell and, eventually, transitional and mature B cell. Such progression is modulated by epigenetic marks, such as DNA methylation and histone posttranslational modifications, which increase chromatin accessibility and target RAG1/RAG2 to V, D, and J DNA (2). It is also dependent on the expression of epigenetic factors, such as microRNAs. In mice deficient in Ago2, which is essential for microRNA biogenesis and function, B cell development is arrested at the pro-B cell stage (Danger et al.). Accordingly, B cell-specific ablation of microRNAs by B cell-specific knockout of Dicer virtually blocks B cell differentiation at the pro-B to pre-B cell transition (Danger et al.).

After mature B cells encounter antigen, changes of the epigenetic landscape are induced by the same stimuli that drive the antibody response. Such epigenetic changes underpin the maturation of the antibody response itself. They instruct those B cell differentiation processes, somatic hypermutation (SHM), class-switch DNA recombination (CSR), and plasma cell differentiation that are central to the maturation of the antibody response as well as the differentiation of memory B cells. Inducible histone modifications, together with DNA methylation and microRNAs, modulate the transcriptome, particularly the expression of activation-induced cytidine deaminase (AID), central to SHM and CSR, and B lymphocyte-induced maturation protein-1 (Blimp-1), the master transcription factor in plasma cell differentiation (1, 3–5) (Shen et al. and Zan and Casali).

Combinatorial histone modifications also function as histone codes in targeting the CSR and, possibly, the SHM machinery to the Ig locus by recruiting specific adaptors (histone code readers) that can in turn target and/or stabilize CSR/SHM factors (6). Epigenetic alterations in memory B cells contribute to their functional distinction from their naive counterparts. Memory B cells inherit epigenetic information from their precursors and acquire new epigenetic marks, which make these resting B cells poised to promptly respond to antigen. The cross/feedback regulation of different epigenetic modifications/elements further increases the complexity of the B cell epigenome, which interacts with the genetic information for precise modulation of gene expression. It is increasingly evident that epigenetic dysregulation in B cells, including aberrant expression of microRNAs, can result in aberrant antibody responses to microbial pathogens, emergence of pathogenic autoantibodies or B cell neoplastic transformation. Epigenetic marks are potential targets for new therapeutics in autoimmunity and B cell malignancy. The collection of experimental and review articles in this research topic addresses from different perspectives the role of epigenetic mechanisms in B cell function and antibody responses.

Zan and Casali presented an overview of the role of epigenetics in CSR, SHM, and B cell differentiation to plasma cells and memory B cells, and therefore, the regulation and maturation of the antibody response. They also highlight our current understanding of epigenetic modulations of CSR, SHM, and plasma cell differentiation by HDAC inhibitors. By performing genome-wide analysis of B cells that are induced to undergo CSR and plasma cells differentiation, Shen et al. further extended their recent discovery showing that HDAC inhibitors modulate the expression of AID and Blimp-1 by regulating the microRNAs that silence *Aicda* and *Prdm1*. This work reveals selective modulation of microRNAs and mRNAs by HDAC inhibitors.

During B cell development, only one allele of the BCR H chain and κ chain or λ chain locus undergoes V(D)J rearrangement at a time, and once productive rearrangement is sensed, rearrangement of the second allele is prevented. Levin-Klein and Bergman summarized recent advances in our understanding of the mechanisms specifying allelic exclusion of antigen receptor genes. They discussed the epigenetic processes, including asynchronous replication, nuclear localization, chromatin condensation, histone modifications, and DNA methylation, which appear to regulate the primary rearrangement of a single allele, while blocking the rearrangement of the second allele.

In immunoglobulins, juxtaposition of the three complementary-determining regions (CDRs) of the L chain and the three of the H chain creates the site that binds antigen. While the CDR1 and the CDR2 are encoded by germline sequences and the CDRL3 is largely so, the CDRH3, which provides the most critical structure basis for antigen binding, is the product of multiple VDJ rearrangements and multiple unencoded N nucleotide insertions. This makes CDRH3 the most diverse component of pre-immune Ig repertoire. The diversity of CDRH3 is constrained by natural selection of Ig D_H_ sequence. Restricted D gene usage leads to defective T-dependent immune responses. By utilizing elegant genetic mouse models and defined B cell response assays, Trad et al. showed that T-dependent B cell responses can be heavily influenced by the effects of natural selection of the D on CDRH3 repertoire diversity. They provided strong evidence that the antigen-independent usage of the D gene segments during VDJ recombination plays a role in modulating the affinity maturation in antigen-dependent B cell responses.

Class-switch DNA recombination is a tightly controlled multistep process involving transcription through switch regions, the DNA cytidine deaminase AID and the participation of several general DNA repair pathways. Vaidyanathan and Chaudhuri reviewed the multilevels of epigenetic regulation that orchestrate CSR, and thoroughly discussed epigenetic controls of switch region accessibility, the epigenetic regulation of AID expression and targeting, as well as of subsequent events of DNA repair.

Expression of AID is tightly regulated due to its mutagenic and recombinogenic potential. AID is known to target not only Ig genes but also non-Ig genes, thereby contributing to lymphomagenesis. In addition to the essential role of AID in CSR and SHM, a new epigenetic function of AID and its link to DNA demethylation has came to light in several developmental systems. Dominguez and Shaknovich summarized existing evidence linking deamination of unmodified and modified cytidine by AID to base-excision repair and mismatch repair machinery resulting in passive or active removal of DNA methylation mark, with the focus on B cell function. They also discuss potential contribution of AID-dependent DNA hypomethylation to B lymphomagenesis.

Transitional B cells (T1 and T2) are selected to avoid self-reactivity and to safeguard against autoimmunity, then differentiate into mature follicular (FO-I and FO-II) and marginal zone (MZ) B cells. To understand how gene expression coordinates transitional B cell tolerance and mature B cell fate, Kleiman et al. performed a comprehensive transcriptome analysis of T1, T2, FO-I, FO-II, and MZ B cell subsets by next-generation RNA-Seq. They identified several genes and gene clusters that are likely linked to specific B cell subsets and transitions of different B cell differentiation stages and their functions.

Understanding the regulation of antibody production and B cell memory formation and function is core to find new treatments for antibody-mediated autoimmune diseases, immunodeficiencies, and B cell-derived cancers. Progression from a small number of antigen-specific B cells to the production of a large number of antibody-secreting cells is tightly regulated. Although much progress has been made in revealing the transcriptional regulation of B cell differentiation that occurs during antibody responses, there are still many questions that remain to be answered. Recent work on the expression and roles of histone modifications in lymphocytes has begun to shed light on this additional level of regulation. Good-Jacobson discussed the recent advancements in understanding how antibody responses, in particular germinal centers and memory B cells, are modulated by histone modifications.

Heterochromatin protein 1γ (HP-1γ) is a highly conserved component of chromatin. It recognizes H3K9 methylation through a chromo-domain and is involved in transcription elongation. *In vitro* observations suggest a role for HP-1γ in the immune system. However, it has not been shown if and how HP-1γ contributes to immunity *in vivo*. Ha et al. uncovered a novel molecular pathway that regulates the adaptive immune response to T-dependent antigens. They demonstrated that HP-1γ positively controls the germinal center reaction and maturation of the high-affinity antibody response by modulating the size of the CD8^+^ T_reg_ compartment.

Eukaryotic cells contain a variety of intracellular membranes, including endosomes, lysosomes, autophagosomes, Golgi network, and endoplasmic reticulum. It is becoming increasingly clear that proteins associated with these membrane compartments play important roles in B cell activation and differentiation. These processes can be regulated by microRNAs, which are important regulators of a wide range of cellular processes. Lou et al. discussed the regulation of B cell differentiation by intracellular membrane-associated proteins and their modulation by microRNAs.

Altogether, these articles provide new and important information for the understanding of how epigenetic marks/factors contribute to B cell development and differentiation, as well as the maturation of the antibody response. We are grateful for the efforts that the authors have made to help us compile this ebook for *Frontiers in Immunology*.

## Author Contributions

HZ and PC worked together to prepare this editorial.

## Conflict of Interest Statement

The authors declare that the research was conducted in the absence of any commercial or financial relationships that could be construed as a potential conflict of interest.
